# Supplementary material for: Existing terminology related to antimicrobial resistance fails to evoke risk perceptions and be remembered
Source: Commun Med (Lond). 2023 Oct 25;3:149. doi: 10.1038/s43856-023-00379-6 (PMC10600229; doi:10.1038/s43856-023-00379-6)
Supplement: Supplementary file 1 — Description of Additional Supplementary Files [file 43856_2023_379_MOESM1_ESM.pdf]

Description of Additional Supplementary Files

File Name: Supplementary Data 1
Description: Number of deaths associated with each health risk term used in the surveys.

File Name: Supplementary Data 2
Description: Source data for Figure 2.

File Name: Supplementary Data 3
Description: Source data for Figure 3.

File Name: Supplementary Data 4
Description: Correlations between risk association, memorability, the different linguistic dimensions and all participant variables for the term "Drug-resistant infections".

File Name: Supplementary Data 5
Description: Correlations between risk association, memorability, the different linguistic dimensions and all participant variables for the term "Antibiotic resistance".
