# Supplementary material for: Existing terminology related to antimicrobial resistance fails to evoke risk perceptions and be remembered
Source: Commun Med (Lond). 2023 Oct 25;3:149. doi: 10.1038/s43856-023-00379-6 (PMC10600229; doi:10.1038/s43856-023-00379-6)
Supplement: Supplementary file 2 — Supplementary information [file 43856_2023_379_MOESM2_ESM.pdf]

Existing Terminology Related to Antimicrobial Resistance Fails to Evoke Risk Perceptions and Be Remembered

Eva M. Krockow, Kate O. Cheng, John Maltby, Eoin McElroy

Supplementary information

Supplementary Note 1

Online survey materials

Page 1: Study Information

Title: Study on health terminology and disease names
Researchers: Our names are [details removed from this document version for double-blinded peer review].
Purpose of data collection: Research on public health risk perception

Details of Participation: This study will take between 10-20 minutes to complete. It's an easy online survey to find out how different people think about different health risks.

First, you'll be required to answer some questions about your personal characteristics (e.g. age and gender). Then you will receive some more instructions for the main task. For the main task, you will have to read a sequence of different words, all of which are names of particular health risks, illnesses or diseases. You will be asked for your personal opinion and experience of these words (e.g. how easy you find them to read and how familiar you are with them). Afterward, you will be given a short word completion task and a brief memory test.

To conclude the survey, the final page will give you more information about the underlying research project and contact details of the researcher in case of further questions.

At the end of the study, you will receive a personal code. It is important that you make a note of this code and enter it into the relevant page on MTURK to confirm your participation and for payments to be processed.

The researchers of this study will be screening responses. Payments will only be awarded to those if we have no doubts that the survey was completed properly.

Page 2: Consent statement

Before the study can begin, you must state whether or not you agree with the consent statement.
Please take some time to read the following information and indicate your acceptance.

1. I understand that my participation is voluntary and that I may withdraw from the research at any time during the survey and up until two weeks after participation by emailing the researcher.
2. I am aware of what my participation will involve.
3. My data are to be held confidentially and only the researchers and close collaborators will have access to them.
4. My data will be kept securely for a period of at least five years after the appearance of any associated publications. Any aggregate data (e.g. spreadsheets) will be kept in electronic form indefinitely but will be anonymous and will not have any identifying information (e.g. names/emails) included on them.
5. In accordance with the requirements of some scientific journals and organisations, my coded data may be shared with other competent researchers. My coded data may also be used in other related studies. My name and other identifying details will not be shared with anyone.
6. The overall findings may be submitted for publication in a scientific journal, or presented at scientific conferences.
7. This study will take approximately 6 months to complete.
8. I will be able to obtain general information about the results of this research by giving the researcher my email address now or by emailing the researcher after study completion.

Please note that this form will be kept separately from your data

If you have further questions about this study, you may contact [details removed from this document version for double-blinded peer review].

I am giving my consent for data to be used for the outlined purposes of the present study.

All questions that I have about the research have been satisfactorily answered.

I have read the following information and:

YES, I do consent.

NO, I do not consent.

In the space below, please now provide details of your MTurk account name to allow for easy identification of your data if you later wish to withdraw from the study.

____

If you would like to receive a summary of the results by e-mail, when this is available, please provide your email address: __________________________________

Page 3: Demographics

1. Please indicate your age: (drop down list of ages 18-100, 100+)

2. Please indicate your sex:
    - Male
    - Female
    - Other
    - Prefer Not to Say

3. Please state your country of residence:

4. Please state your nationality:

5. Please state your race:
    - White American
    - Hispanic and Latino Americans (of any race)
    - Black or African American
    - Asian American
    - Native Americans and Alaska Natives
    - Native Hawaiians and Other Pacific Islanders
    - Two or more races
    - Other (if you selected Other, please specify)

6. Highest education Level:
    - high school
    - community college/junior college/vocational technical institute
    - Bachelor’s degree
    - Master’s degree
    - PhD
    - Other

7. Please state the name of your First/ Native Language:

8. If applicable, please now state any additional native languages you are fluent in:

9. What statement best describes your current employment status?

    - working full time
    - working part time
    - not working
    - prefer not to say

10. Which of the following industries most closely matches the one in which you are employed?:
    - Retail trade
    - Wholesale trade
    - Health care or social assistance
    - Professional, scientific or technical services
    - Finance or insurance
    - Transportation or warehousing
    - Information
    - Admin, support, waste management or remediation services
    - Management of companies or enterprises

- Arts, entertainment or recreation
- Manufacturing
- Mining
- Forestry, fishing, hunting or agriculture support
- Real estate or rental and leasing
- Educational services
- Accommodation or food services
- Construction
- Utilities

Page 4: Medical History

1. Have you visited a doctor in the past 12 months?

- Yes
- No
- I don’t know
- Prefer not to say

2. If yes, roughly, how many times in the past 12 months have you visited a doctor?

3. Have you taken antibiotics within the past 12 months?

- Yes
- No
- I don’t know
- Prefer not to say

Page 5: Task 1 Instructions

On the following pages, you will be presented with 42 words, all of which refer to a health threat, illness or disease.

Using the measures below, you will be required to rate each of these based on your personal experiences and opinions of the words. Please note, there is no right or wrong answer here; we are interested in your own views and thoughts.

Please read the following information about rating scales carefully.
You will be given reminders of the scales and measures when required to rate the words.

Familiarity: Please rate how familiar the word seems to you using the scale below. For example, if you consider the word to be a well-known part of your regular vocabulary, you would give it a rating of “very familiar”. If you can’t remember ever having seen or heard the word, you would give it a rating of “very unfamiliar”.

- Very unfamiliar
- Moderately unfamiliar
- Somewhat unfamiliar
- Not sure
- Somewhat familiar
- Moderately familiar
- Very familiar

Processing fluency: Please rate the ease or difficulty of reading and understanding the word using the scale below. For example, if you can read and understand the word easily without having to concentrate, please give it a rating of “very easy”. If you struggle to read and understand word (even when concentrating), please give it a rating of “very difficult”.

- Very difficult
- Moderately difficult
- Somewhat difficult
- Not sure
- Somewhat easy
- Moderately easy
- Very easy

Pronounceability: Please rate the ease or difficulty of pronouncing the word if you were to read it aloud, again using the scale below. For example, if you find it very easy to pronounce the word, please give it a rating of “very easy”. If you don’t know how to pronounce the word or struggle to read it aloud, please give it a rating of “very difficult”.

- Very difficult
- Moderately difficult
- Somewhat difficult
- Not sure
- Somewhat easy
- Moderately easy
- Very easy

Concreteness: Please rate the concreteness of the word using the scale below. For example, if you feel like a word refers to a real, tangible object, material, or person (e.g., house, hand), please give it a rating of “very concrete”. If you find the word very abstract, referring to ideas and concepts that cannot be directly experienced by the senses (e.g., risk, proof), please give it a rating of “very abstract”.

- Very abstract
- Moderately abstract
- Somewhat abstract
- Not sure
- Somewhat concrete
- Moderately concrete
- Very concrete
-

Riskiness: Please rate the risk you associate with the word using the scale below. For example, if you feel like the word describes a condition that is generally highly risky and dangerous, please give it a rating of “Very risky”. If you feel like the word describes a condition that generally isn’t risky or dangerous at all, please give it a rating of “Very safe”.

- Very risky
- Moderately risky
- Somewhat risky
- Not sure
- Somewhat safe
- Moderately safe
- Very safe

Please confirm you have read and understood the instructions above to proceed to the task.

I have read and understood the instructions

Pages 6-48: Presentation of 40 health-related terms

The following word stimuli were presented on separate pages in randomised orders. The two filler items were inserted in position 17 (“table”) and position 32 (“school”).

1. AIDS
2. Air Pollution
3. Alzheimer’s
4. AMR
5. Anaemia
6. Antibiotic resistance
7. Antimicrobial resistance
8. Asthma
9. Bacterial resistance
10. Cancer
11. Chicken Pox
12. Climate Change
13. Coronavirus
14. COVID-19
15. Dengue
16. Depression
17. Diabetes
18. Diarrhoea
19. Drought
20. Drug resistant infections
21. Ebola
22. Famine
23. Gonorrhoea
24. Heart disease
25. High Blood Glucose
26. High Cholesterol
27. HIV
28. Influenza
29. Malaria
30. Measles
31. Meningitis
32. Obesity
33. Parkinson’s
34. Pneumonia
35. Salmonellosis
36. SARS
37. Schizophrenia
38. Superbugs
39. Tuberculosis
40. Zika

[WORD STIMULUS]

Please rate the following word on the five scales below:
Full definitions of each rating scale are presented at the bottom of the page.

Familiarity:
- Very unfamiliar
- Moderately unfamiliar
- Somewhat unfamiliar
- Not sure
- Somewhat familiar
- Moderately familiar
- Very familiar

Processing fluency:
- Very difficult
- Moderately difficult
- Somewhat difficult
- Not sure
- Somewhat easy
- Moderately easy
- Very easy

Pronounceability:

- Very difficult
- Moderately difficult
- Somewhat difficult
- Not sure
- Somewhat easy
- Moderately easy
- Very easy

Concreteness:

- Very abstract
- Moderately abstract
- Somewhat abstract
- Not sure
- Somewhat concrete
- Moderately concrete
- Very concrete

Riskiness:

- Very risky
- Moderately risky
- Somewhat risky
- Not sure
- Somewhat safe
- Moderately safe
- Very safe

Familiarity: Please rate how familiar the word seems to you. For example, if you consider the word to be a well-known part of your regular vocabulary, you would rate it as “very familiar”. If you can’t remember ever having seen or heard the word, you would rate it as “very unfamiliar”.

Processing fluency: Please rate the ease or difficulty of reading and understanding the word using the scale below. For example, if you can read and understand the word easily without having to concentrate, please give it a rating of “very easy”. If you struggle to read and understand word (even when concentrating), please give it a rating of “very difficult”.

Pronounceability: Please rate the ease or difficulty of pronouncing the word if you were to read it aloud, again using the scale below. For example, if you find it very easy to pronounce the word, please give it a rating of “very easy”. If you don’t know how to pronounce the word or struggle to read it aloud, please give it a rating of “very difficult”.

Concreteness: Please rate the concreteness of the word using the scale below. For example, if you feel like a word refers to a real, tangible object, material, or person (e.g., house, hand), please give it a rating of “very concrete”. If you find the word very abstract, referring to ideas and concepts that cannot be directly experienced by the senses (e.g., risk, proof), please give it a rating of “very abstract”.

Riskiness: Please rate the risk you associate with the word using the scale below. For example, if you feel like the word describes a condition that is generally highly risky and dangerous, please give it a rating of “Very risky”. If you feel like the word describes a condition that generally isn’t risky or dangerous at all, please give it a rating of “Very safe”.

Page 49: Task 2

We would now like you to complete a short vocabulary task.

In the corresponding boxes below, please give a brief description of each word. For example, for the word “scarf”, you could write: “item of clothing worn around the neck”.

Alternatively, you could write down a synonym (i.e., a word that has a very similar meaning). For example, for the word “loud”, you could write the synonym: “noisy”.

You will be given points based on how good your description of each word is.

1. Apple
2. Glove
3. Breakfast
4. Curious
5. Assemble
6. Consume
7. Terminate
8. Ponder
9. Reluctant
10. Confide
11. Remorse
12. Plagiarise
13. Acute
14. Generate
15. Compassion
16. Tangible
17. Evolve
18. Diverse
19. Fortitude
20. Ominous
21. Encumber
22. Audacious
23. Tirade
24. Pragmatic
25. Palliate

Page 50: Task 3

We'd like to now ask you to recall as many of the words concerning health risks, illnesses or diseases that you can remember from the first, Task 1, of the study.

Please recall and type out as many of the words that you can remember in the space below.

Page 51: Task 4

Similar to the task before, please now indicate whether or not you recall seeing the following words from the first, Task 1, of the study.

Please use the multiple choice answers and state whether or not you recall seeing the following words.

YES, I remember seeing this word

NO, I do not remember seeing this word

1. Psychosis
2. Acne
3. Vaccination
4. Dementia
5. Heart attack
6. Epilepsy
7. Multiple Sclerosis
8. Addison's disease
9. Appendicitis
10. Arthritis
11. Sepsis/ Septicaemia
12. Bronchitis
13. Anxiety
14. Coeliac disease
15. Cystic fibrosis
16. Constipation
17. Dizziness
18. Eczema
19. Endometriosis
20. Hepatitis
21. Insomnia
22. Migraine
23. Obsessive compulsive disorder
24. Psoriasis
25. STI's
26. Shingles
27. Stroke
28. Conflict
29. Tonsillitis
30. UTI
31. Yellow Fever
32. Scarlet Fever
33. Tinnitus
34. Global Warming
35. Malnutrition
36. Poverty
37. Fever
38. Sickle cell disease
39. Mental Health
40. Osteoporosis
41. AMR
42. Cancer
43. COVID-19
44. Malaria
45. Anaemia
46. High Cholesterol
47. Drug resistance infections
48. Influenza
49. Coronavirus
50. Famine
51. SARS
52. Schizophrenia
53. Asthma
54. Salmonellosis
55. Alzheimer's
56. Diabetes
57. Pneumonia
58. AIDS
59. Obesity
60. Parkinson's
61. Diarrhoea
62. Gonorrhoea
63. Meningitis
64. Chicken Pox
65. Drought
66. Zika
67. Measles
68. Antibiotic resistance
69. Superbugs
70. Antimicrobial resistance
71. Depression
72. Air Pollution
73. Ebola
74. HIV
75. Bacterial resistance
76. High Blood Glucose
77. Tuberculosis
78. Climate Change
79. Heart disease
80. Dengue

Page 52: Final page

You have now finished the survey.

Thank you for taking the time to complete this survey.

Study Debrief

The research is being conducted to investigate how people think about and view different health risks with the aim that this knowledge can contribute towards better and more successful science communication with lay audiences. More specifically, we asked you to rate various health-risk related words on their processing fluency, pronounceability, familiarity, concreteness and risk - all of which are known factors that have been shown to affect the way in which audiences may process and think about different words. Furthermore, memory of these words was assessed at the end of the study to test whether the terminology of public health risks affects how well people retain the associated information.

Additionally, the study aimed to investigate the effect of the different terms describing 'Antimicrobial resistance' as there is a lack of consistency amongst science communication for the term. For example, amongst media and communication with lay audiences, there are multiple commonly used terms, which describe antimicrobial resistance. These include 'antibiotic resistance', 'AMR', 'superbugs' and 'drug-resistant infections'. We hypothesise that such inconsistencies in terms, may contribute towards the lack of engagement by the media and non-scientific audiences in recognising antimicrobial resistance as a global health threat.

For more information about Antimicrobial resistance, please visit the following links:

World Health Organisation: https://www.who.int/health-topics/antimicrobial-resistance

Supplementary Table 1.

Study 1 ANOVA comparing mean risk association ratings for the 6 AMR-related health terms: Pairwise comparisons with Bonferroni adjustment for multiple comparisons

Risk association	Risk association	Mean Difference	Std. Error	Sig.[b]	95% Confidence Interval for Difference[b] Lower Bound	Upper Bound
AMR	Antibiotic Resistance	-.928*	.100	<.001	-1.223	-.633
	Antimicrobial Resistance	-.494*	.098	<.001	-.785	-.202
	Bacterial Resistance	-.574*	.110	<.001	-.901	-.247
	Drug Resistant Infections	-1.249*	.100	<.001	-1.545	-.953
	Superbugs	-.861*	.101	<.001	-1.161	-.561
Antibiotic Resistance	AMR	.928*	.100	<.001	.633	1.223
	Antimicrobial Resistance	.435*	.095	<.001	.152	.717
	Bacterial Resistance	.354*	.102	.009	.052	.657
	Drug Resistant Infections	-.321*	.087	.004	-.579	-.062
	Superbugs	.068	.087	1.000	-.190	.325
Antimicrobial Resistance	AMR	.494*	.098	<.001	.202	.785
	Antibiotic Resistance	-.435*	.095	<.001	-.717	-.152
	Bacterial Resistance	-.080	.088	1.000	-.341	.180
	Drug Resistant Infections	-.755*	.099	<.001	-1.050	-.461
	Superbugs	-.367*	.103	.007	-.672	-.062
Bacterial Resistance	AMR	.574*	.110	<.001	.247	.901
	Antibiotic Resistance	-.354*	.102	.009	-.657	-.052
	Antimicrobial Resistance	.080	.088	1.000	-.180	.341
	Drug Resistant Infections	-.675*	.105	<.001	-.986	-.364
	Superbugs	-.287	.104	.092	-.595	.021
Drug Resistant Infections	AMR	1.249*	.100	<.001	.953	1.545
	Antibiotic Resistance	.321*	.087	.004	.062	.579
	Antimicrobial Resistance	.755*	.099	<.001	.461	1.050
	Bacterial Resistance	.675*	.105	<.001	.364	.986
	Superbugs	.388*	.092	<.001	.115	.662
Superbugs	AMR	.861*	.101	<.001	.561	1.161
	Antibiotic Resistance	-.068	.087	1.000	-.325	.190
	Antimicrobial Resistance	.367*	.103	.007	.062	.672
	Bacterial Resistance	.287	.104	.092	-.021	.595
	Drug Resistant Infections	-.388*	.092	<.001	-.662	-.115

Based on estimated marginal means

*. The mean difference is significant at the .05 level.

b. Adjustment for multiple comparisons: Bonferroni.

Supplementary Table 2.

Study 2 ANOVA comparing mean risk association ratings for the 6 AMR-related health terms: Pairwise comparisons with Bonferroni adjustment for multiple comparisons

					95% Confidence Interval for Difference[b]	
Risk association	Risk association	Mean Difference	Std. Error	Sig.b	Lower Bound	Upper Bound
AMR	Antibiotic Resistance	-1.555*	.044	<.001	-1.684	-1.427
	Antimicrobial Resistance	-.800*	.043	<.001	-.926	-.674
	Bacterial Resistance	-1.008*	.048	<.001	-1.148	-.867
	Drug Resistant Infections	-1.750*	.044	<.001	-1.879	-1.621
	Superbugs	-1.470*	.045	<.001	-1.603	-1.336
Antibiotic Resistance	AMR	1.555*	.044	<.001	1.427	1.684
	Antimicrobial Resistance	.755*	.046	<.001	.619	.891
	Bacterial Resistance	.548*	.045	<.001	.415	.680
	Drug Resistant Infections	-.195*	.039	<.001	-.309	-.081
	Superbugs	.085	.045	.878	-.047	.218
Antimicrobial Resistance	AMR	.800*	.043	<.001	.674	.926
	Antibiotic Resistance	-.755*	.046	<.001	-.891	-.619
	Bacterial Resistance	-.208*	.043	<.001	-.335	-.080
	Drug Resistant Infections	-.950*	.047	<.001	-1.088	-.812
	Superbugs	-.670*	.048	<.001	-.810	-.530
Bacterial Resistance	AMR	1.008*	.048	<.001	.867	1.148
	Antibiotic Resistance	-.548*	.045	<.001	-.680	-.415
	Antimicrobial Resistance	.208*	.043	<.001	.080	.335
	Drug Resistant Infections	-.742*	.048	<.001	-.882	-.603
	Superbugs	-.462*	.049	<.001	-.606	-.318
Drug Resistant Infections	AMR	1.750*	.044	<.001	1.621	1.879
	Antibiotic Resistance	.195*	.039	<.001	.081	.309
	Antimicrobial Resistance	.950*	.047	<.001	.812	1.088
	Bacterial Resistance	.742*	.048	<.001	.603	.882
	Superbugs	.280*	.044	<.001	.152	.409
Superbugs	AMR	1.470*	.045	<.001	1.336	1.603
	Antibiotic Resistance	-.085	.045	.878	-.218	.047
	Antimicrobial Resistance	.670*	.048	<.001	.530	.810
	Bacterial Resistance	.462*	.049	<.001	.318	.606
	Drug Resistant Infections	-.280*	.044	<.001	-.409	-.152

Based on estimated marginal means

*. The mean difference is significant at the .05 level.

b. Adjustment for multiple comparisons: Bonferroni.

Supplementary Table 3.

Study 1 Cochran's Q test comparing memorability scores for the 6 AMR-related health terms: Pairwise comparisons with Bonferroni adjustment for multiple comparisons

Sample 1-Sample 2	Test Statistic	Std. Error	Std. Test Statistic	Sig.	Adj. Sig.[a]
Antibiotic resistance – Bacterial resistance	-.030	.028	-1.066	.286	1.000
Antibiotic resistance - Superbugs	-.051	.028	-1.827	.068	1.000
Antibiotic resistance - Antimicrobial resistance	-.051	.028	-1.827	.068	1.000
Antibiotic resistance - Drug resistant infections	-.111	.028	-3.959	<.001	.001
Antibiotic resistance - AMR	-.111	.028	-3.959	<.001	.001
Bacterial resistance - Superbugs	.021	.028	.761	.446	1.000
Bacterial resistance - Antimicrobial resistance	-.021	.028	-.761	.446	1.000
Bacterial resistance - Drug resistant infections	.081	.028	2.893	.004	.057
Bacterial resistance- AMR	.081	.028	2.893	.004	.057
Superbugs - Antimicrobial resistance	.060	.028	2.132	.033	.496
Superbugs- Drug resistant infections	.060	.028	2.132	.033	.496
Superbugs - AMR	.000	.028	.000	1.000	1.000
Antimicrobial resistance - Drug resistant infections	-.060	.028	-2.132	.033	.496
Antimicrobial resistance - AMR	.060	.028	2.132	.033	.496
Drug resistant infections - AMR	.000	.028	.000	1.000	1.000

Each row tests the null hypothesis that the Sample 1 and Sample 2 distributions are the same.
Asymptotic significances (2-sided tests) are displayed. The significance level is .050.
a. Significance values have been adjusted by the Bonferroni correction for multiple tests.

Supplementary Table 4.

Study 2 Cochran's Q test comparing memorability scores for the 6 AMR-related health terms: Pairwise comparisons with Bonferroni adjustment for multiple comparisons

Sample 1-Sample 2	Test Statistic	Std. Error	Std. Test Statistic	Sig.	Adj. Sig.[a]
Antibiotic resistance – Bacterial resistance	-.021	.015	-1.377	.168	1.000
Antibiotic resistance - Superbugs	-.031	.015	-2.102	.036	.533
Antibiotic resistance - Antimicrobial resistance	-.078	.015	-5.220	<.001	.000
Antibiotic resistance - Drug resistant infections	-.088	.015	-5.872	<.001	.000
Antibiotic resistance - AMR	.101	.015	6.742	<.001	.000
Bacterial resistance - Superbugs	-.011	.015	-.725	.468	1.000
Bacterial resistance - Antimicrobial resistance	.057	.015	3.842	<.001	.002
Bacterial resistance - Drug resistant infections	-.067	.015	-4.495	<.001	.000
Bacterial resistance- AMR	.080	.015	5.365	<.001	.000
Superbugs - Antimicrobial resistance	.047	.015	3.117	.002	.027
Superbugs- Drug resistant infections	.056	.015	3.770	<.001	.002
Superbugs - AMR	.069	.015	4.640	<.001	.000
Antimicrobial resistance - Drug resistant infections	-.010	.015	-.652	.514	1.000
Antimicrobial resistance - AMR	.023	.015	1.522	.128	1.000
Drug resistant infections - AMR	.013	.015	.870	.384	1.000

Each row tests the null hypothesis that the Sample 1 and Sample 2 distributions are the same.
Asymptotic significances (2-sided tests) are displayed. The significance level is .050.
a. Significance values have been adjusted by the Bonferroni correction for multiple tests.
